# New Standards and Old

**Published:** 1915-06

**Authors:** 


### NEW STANDARDS AND OLD.

Not many years ago the public drinking cup, the common milk can, the unquestioned oyster, were articles of established utility and repute in the most discriminating community. Those were the golden days when most human ills might be referred to an inscrutable Providence, and man was permitted to suffer his self-inflicted troubles with a clean conscience. A contrariwise disposition, however, has since impelled him to "investigate" many former conveniences and comforts, with the result of finding that he must forego or "regulate" them—under penalty of having only himself to blame for consequences. This leaves him perhaps not happier, but wiser, and, barring accidents, likely to live the longer.

Frequently we have pleaded the importance of remembering what is good in the old before making radical change for what is new. It is so easy to forget the hard lessons of experience in the enthusiasm which warms a new idea. But it is even more important to see the good—the really good—in the new, in a world which must progress. True progress is like ascending a winding stair in a tower: at each window we see the same picture, but wider and forever different, because we see so much the farther. The same elements exist—but the new horizon brings a new adjustment of proportions: we can not see them as at first, except by shutting our eyes to the light.

Among recent changes in operative dentistry, that of improved root canal work is undoubtedly the most important. It is a truism to say a tooth is no better than its root. And now we know that a diseased root not only means a painful and useless organ, but a menace to the general health of the patient of so serious a nature as to require prompt cure or removal in all cases. The "comfortable" chronic abscess of old is a potent factor in the causation of many acute systemic troubles, and in organic injuries leading to premature senility. It must be eradicated; and, as extraction means a serious loss in most cases, successful root work becomes of commanding importance.

No operator, however skillful, can do correct root work without the roentgenogram: the best of operators are being daily amazed and humiliated with the truth in inspecting their old work by this means. Knowledge, instead of surmise, concerning the length and shape of roots, is essential in this work, and the X-ray is the only means available.

The old dread of ''going through'' at the apical foramen must be supplanted by the determination on the part of the operator to do exactly that in all cases. Anything short of reaching healthy periapical tissue implies leaving a zone of dead material of greater or less thickness at the root end, which may at any time of lowered resistance on the part of the patient recruit a bacterial army of invasion. The cleansing of the canal to its ultimate end is necessary, and this means aseptic instrumentation and the aseptic filling of that canal. The sterile gutta percha filling, penetrating and ''encapsulating'' the apex, advocated by Rhein, seems the most dependable method available at this time.

The metallic sodium and potassium paste is far safer than sulfuric acid as a means of tracing and opening obliterated canals. This paste attacks only the organic matrix of the dentin, and by its destruction and removal the way is cleared for the broach, whereas the acid method involves the dissolution of the inorganic as well as organic constituents, with the frequent result that a ''false pocket,'' once formed, is easily carried to a perforation at the side of the root.

Where it is mechanically impossible to cleanse a crooked canal, amputation of the unfilled end is the surest means of safety. Where this is impracticable, thorough sterilization of dentin by ionism may prevent future septic conditions; but of this there can not be certainty. Ionism is an excellent final precaution in all root canal work, but should not take the place of perfect filling.

We have not reached perfection in technique, but we have learnt the necessity of thorough root work, and its feasibility, though tedious and difficult. In the light of present knowledge, we must meet the new and heavy responsibility which rests upon every dental practitioner.—Editorial in March *Journal of Allied Societies.*

